# Hematological characteristics and hepatobiliary complications of hereditary spherocytosis in a tertiary care pediatric center: optimizing diagnosis and care through local and international networks

**DOI:** 10.3389/fped.2023.1269645

**Published:** 2023-10-09

**Authors:** Maria Paola Boaro, Giulia Reggiani, Mirco D’Agnolo, Vania Munaretto, Francesco Pozzebon, Roberta Trapanese, Maddalena Martella, Raffaella Colombatti

**Affiliations:** ^1^ UOC Pediatric Hematology Oncology, Azienda Ospedale Università di Padova, Padova, Italy; ^2^ Department of Women’s and Child’s Health, University of Padova, Padova, Italy

**Keywords:** spherocytosis, hereditary, diagnosis, biliary, spleen, children

## Abstract

**Background:**

Hereditary Spherocytosis (HS) is a rare, congenital red blood cell disorder presenting with variable clinical manifestations ranging from mild hemolytic anemia to severe anemia with hypersplenism and hepatobiliary complications.

**Methods:**

The objectives of the study were to evaluate the diagnostic pathway of HS, the presence and management of hepatobiliary complications in pediatric patients with HS followed in a tertiary care center. The demographic, clinical, hematological information were retrieved from medical records of patients having at least 1 hematology visit between 2010 and 2020.

**Results:**

Forty-two patients were enrolled, 23 M. Mean age at onset of symptoms was 2.8 years, at diagnosis was 3.5 years. Anemia was the first manifestation in 73%; suspect of HS arose for all patients in first or second level outpatient clinics. Only 64% of patients performed two confirmation tests in the reference center. 28/42 had familiarity for HS; of the 13/42 who did not, only 47% performed further analysis. Sixteen patients developed gallbladder stones (40%), visible at the first ultrasound (5.6 years). Hemolytic crises and parvovirus infections were more frequent in patients with stones (53.6% vs. 26.1% and 63.6% vs. 28.6%, respectively). 10/16 (62.5%) underwent elective cholecystectomy: 8 had concomitant splenectomy.

**Conclusions:**

our study highlights the need to optimize the diagnostic pathway in networks of care involving general and specialized centers in order to reduce time to diagnosis and ensure that all patients receive confirmatory tests. A high frequency of hepatobiliary complications since young age was observed suggesting that screening with ultrasound should begin earlier

## Introduction

Hereditary Spherocytosis (HS) is the most common hereditary hemolytic anemia in Northern Europe and North America, where its estimated prevalence is 1:1,000–1:2,000 (1). It is a rare genetic disease, caused by abnormalities of the red cell cytoskeleton that increases the osmotic fragility, due to isolated or combined proteins deficiency or disfunction (spectrin, ankyrin, band 3, protein 4.2) (2) leading to chronic hemolytic anemia. Spherocytic cells, which are a hallmark of the disease, have reduced deformability and are subject to increased phagocytosis in the spleen. Anemia can worsen during infections, due to hemolytic crisis, or aplastic crisis, as in case of Parvovirus B19; megaloblastic crisis can be rarely seen with B12 vitamin and folic deficiencies. Red blood cell transfusions or erythropoietin might be necessary in the neonatal period and the first year of life due to the reduced capacity of medullar erythropoiesis to compensate for the rate of hemolysis (3, 4).

Diagnostic pathway for HS usually includes the presence of family and clinical history, physical examination with jaundice and enlargement of the spleen, presence of chronic hemolysis (increased indirect bilirubin, reticulocyte count and lactate dehydrogenase, decreased haptoglobin with or without anemia), and characteristic red blood cell morphology (spherocytes) (5, 6). According to the 2011 laboratory guidelines, positivity to two specific tests is needed. Osmotic fragility test (OFT) and the eosin-5′-maleimide binding test (EMA binding test) are the most used ones by reference laboratories; OFT has limited sensitivity and specificity, and approximately 20% of mild cases of HS remained undiagnosed using only this test (1); EMA binding provides excellent sensitivity and specificity of >86% and often even >95% (7). Nevertheless, around 5%–10% of patients remain undiagnosed due to normal EMA binding. Alternatively, the second test that can be used are the pink test or the acidified glycerol lysis test (AGLT), with a sensitivity of 95% and 91% respectively (7).

The gold standard for the measurement of red blood cell deformability is the osmotic gradient ektacytometry, but unfortunately the instrument is available only in few laboratories (8).

Confirmation of the defective protein is usually performed with the sodium dodecyl sulphate-polyacrylamide gel electrophoresis (SDS-PAGE), in selected cases such those in which the screening tests are equivocal (5). Genetic analyses are, at the moment, performed only in specific situations such as negative family history, or normal EMA binding test. Recent findings show that clinical variability, severity and evolution during the lifespan can vary according to the genotype, hence, new genetic technologies (i.e., Next Generation Sequencies) could enter, in the next future, in the diagnostic pathway (9, 10).

Clinical phenotype can be very different among patients, ranging from mild anemia, diagnosed only in adult age, to very severe clinical manifestations since the neonatal period.

The most frequent chronic complications are cholelithiasis and splenomegaly with hypersplenism, less frequent events are choledochal lithiasis and recurrent cholecystitis which usually occur before 13 years of age; most of the patients remain without symptoms, even if in some cases they show acute and chronic complications since infancy (11, 12). Nevertheless, abdomen ultrasound is suggested annually, after 5 years of age (5). Consensus among experts has been reached if patients present symptoms of gallbladder stones, in which case cholecystectomy is indicated; on the contrary, no clear indication are defined on the on management of asymptomatic lithiasis and cholecystectomy is not recommended in asymptomatic patients who undergo to splenectomy while splenectomy is generally not recommended only to reduce lithiasis complications. HS is a rare hematological disease, and the most recent guidelines to which we can refer are the “Guidelines on hereditary spherocytosis” (5) and the guidelines of the European Hematology Association (EHA) on splenectomy in children (13).

In recent years, the need to create networks for sharing knowledge about rare diseases has arisen. In 2011, the European Union strongly wanted to create experts' groups based on patients' rights in cross-border healthcare (14), of which the first expression was European Reference Networks (ERN). EuroBloodNet is the ERN for rare hematologic diseases, endorsed in 2017. One of its groups, in which spherocytosis is included, is mainly interested in red blood cell diseases and contributed to disseminate diagnostic protocols among all participating centers.

The aim of our study was to review the diagnostic pathway, the clinical characteristics, and the therapeutic approach of HS patients followed in the Pediatric Hematology and Oncology Unit of Padova University, with a special focus on biliary complications.

## Materials and methods

### Setting

The Pediatric Hematology Oncology Unit is the Veneto Region hub center for oncologic and hematological diseases of the pediatric age, in North East Italy, officially recognized since 2014. A network of pediatric spoke centers involved in first and second level of care is present within the Region. Protocols of diagnosis and care are shared within the network, although regular meetings are held more for oncological diseases than for rare hematological benign disorders.

Routine follow-up protocol for HS at our institution consists of at least one hematology visit per year and annual laboratory exams and abdominal ultrasound (more in case of clinical needs); the latter can be performed in a general clinic or regional hospital and visualized during the annual hematology visit.

### Patient population

In order to describe the diagnostic pathway, clinical and laboratory characteristics of our population, data on HS patients followed at our center were retrospectively collected. Patients with at least one follow-up hematology visit between 2010 and 2020 at the reference center were included for homogeneity of diagnosis and follow-up. Medical history (which included also all the reports from the sending pediatricians in the general hospitals), physical examination during the hematology visit and laboratory tests were extracted from the patients' clinical charts on a dedicated excel file for analysis. Acute clinical complications (hemolytic or aplastic crises) and chronic complications (cholecystitis, cholelithiasis, cholecystectomy and splenectomy) were also extracted from clinical charts.

Indication for splenectomy during the study period was according to the international guidelines, for patients who were transfusion dependent or with severe anemia (5, 6, 13). Indication for cholecystectomy was according to acute complications; no clear indication was present in case of asymptomatic gallbladder stones (5, 13).

Between 2010 and 2020, first level tests (full blood count, blood smear evaluation) were available in all spoke centers; second level tests' availability was generally more limited: OFT was available in our center while EMA binding test was performed in our institution starting in 2017; previously, samples had to be sent to another Region, to the Ospedale Maggiore Policlinico of Milan, after authorization from the hospital's direction. Quantitative analysis of membrane protein (SDS-PAGE) was also performed in Ospedale Maggiore Policlinico of Milan.

### Statistical analysis

Descriptive statistic was used to organize and present data. Statistical calculations were made by SAS software 9.4 (SAS Institute Inc., Cary, NC, USA) for Windows. Q-Q plot test and Shapiro–Wilk tests were used to explore the normality of quantitative variables. When comparing two groups of patients (age at diagnosis <1 year, 1–2 years, 2–3 years, >3 years, familiar history present/absent, defective membrane protein, presence of cholelithiasis, cholecystectomy) with respect to quantitative variables, the Mann–Whitney test; for more than two groups Kruskall–Wallis test was used. For categorical variables, the groups were compared with Fisher's exact test.

## Results

Forty-two out of 84 HS patients followed in our Reference Center had at least one follow-up hematology visit between 2010 and 2020 and were therefore included in the analysis. All the patients were first evaluated in a spoke hospital near home where they received first suspect of HS; they were then sent for diagnosis confirmation to the reference hub center. All the patients showed up for the hematology visit and then returned to their centers.

Nineteen females (45%) and 23 males were included in the study. Five sibling pairs and 1 couple of twin sisters were in the group. A total of 28/41 (68.3%) patients had family history of HS. Mean age at study analysis was 12 years and 9 months ±5 years and 7 months.

### Family history and diagnosis timing

Forty-one patients had information on family history: 28 (68.3%) had one relative affected by HS, 13 had a negative family history.

Symptoms related to spherocytosis were recorded in the charts to have appeared at a mean age of 2 years and 10 months (±3 years and 8 months), with a median of 1 year and 5 months. However, 27/42 (64%) of the medical records from regional general hospitals had information on neonatal period: 17 patients had jaundice in the neonatal period (3 required phototherapy), 6 patients had severe anemia and were transfused during the first month of life, 4 presented both jaundice and severe anemia and 2 of them were transfused during the first month of life.

The mean age at diagnosis was 3 years and 6 months (±3 years and 10 months), with a median of 1 year and 10 months. Mean time from first symptoms to diagnosis confirmation was 6 months (±12 months). Almost half of the patients (42.8%) received diagnosis after the age of 3 years, 33% were diagnosed above 1 year. Time to diagnosis was longer in patients with family history, even if no statistical differences was found (mean 199.13 ± 453.94 vs. 165.00 ± 168.78 days).

Anemia was reported to be the first manifestation of the disease in 27/37 (73%) patients, 11 of which had hemoglobin lower than 8 g/dl. Thirty-seven out of 42 patients had complete information on the results of the diagnostic tests in the charts. 10/37 (27%) asymptomatic patients performed blood tests due to the presence of an affected relative.

### Diagnosis

First level laboratory tests were performed for all patients in the spoke centers of the Pediatric Hematology-Oncology Network (complete blood count, hemolytic variables). Hematological variables according to age at diagnosis are shown in Table 1.

**Table 1:** Hematological parameters according to age at diagnosis.

Variable	Total (Mean ± SD) *N* = 35*	<1 years (Mean ± SD) *N* = 12	1–2 years (Mean ± SD) *N* = 6	>2–3 years (Mean ± SD) *N* = 2	>3 years (Mean ± SD) *N* = 15
Red blood cells (10^12^/L)	3.50 ± 0.81	3.22 ± 0.72	3.48 ± 0.82	3.85 ± 0.21	3.66 ± 0.92
Hemoglobin (g/L)	101.83 ± 25.44	104.08 ± 29.69	96.50 ± 23.46	112.50 ± 10.61	100.73 ± 25.30
Hematocrit (%)	29.48 ± 6.90	30.78 ± 6.80	28.68 ± 7.77	30.95 ± 0.64	28.50 ± 7.42
Reticulocytes (cells/ul)	284.18 ± 177.49	292.31 ± 233.67	237.30 ± 168.35	258.00**	301.95 ± 156.46
Total bilirubin (mg/dl)	6.70 ± 9.90	15.31 ± 15.96	3.61 ± 3.63	1.75 ± 0.92	3.52 ± 2.28
gGT (U/L)	51.70 ± 50.31	74.50 ± 88.39	80.00 ± 11.31	N/A	34.67 ± 45.73
ALP (U/L)	222.50 ± 20.51	208.00	237.00	N/A	
LDH (U/L)	571.50 ± 380.52	421.00 ± 170.54	869.50 ± 458.91	602.00**	587.25 ± 470.74
Aptoglobin (mg/dl; n.v. 30–200)	12.65 ± 14.05	24.00 ± 27.71	11.67 ± 6.35	N/A	7.47 ± 0.90
AST (U/ml)	39.00 ± 15.07	38.75 ± 12.04	45.33 ± 21.73	35.50 ± 3.54	37.90 ± 16.79
Ferritin (ng/ml)	457.18 ± 494.90	654.95 ± 387.48	729.85 ± 995.82	52.00 ± . (*N* = 1)	146.77 ± 44.04

* For 7 patients the first complete blood count was not available.

** Data for only one patient was available.

Blood film morphology was evaluated in 37 patients at diagnosis, and in 36 of them spherocytes were found; in the remaining one, spherocytes were found during a second control.

Information on the results of the confirmation tests was available for 37/42 patients. Only 24/37 HS patients (64.9%) had the results of both tests recommended by the Guidelines. However, all patients diagnosed after 2017 when EMA binding test became available locally, had both tests performed. Thirty-two patients had OFT (26 of them resulted positive, 6 negative); 28 patients had EMA binding test (27 positive, 1 negative). 13/37 patients (25**.**1%) had only one confirmatory test, of whom in a total of 2 cases only SDS-PAGE was performed.

Seventeen patients performed also SDS PAGE, to quantify the membrane protein defect. 10/17 had a familiar history positive for HS. Only 7 of 13 patients who did not have familiar history, performed the SDS PAGE test. Ten patients had a single protein defect (8 spectrin, 1 band 3, 1 ankirine) while 7 had combined defects.

### Acute events and biliary complications

Twelve patients presented an aplastic crisis due to Parvovirus B19 and 11 patients presented hemolytic crisis requiring transfusion after the first year of life.

**Figure 1:**
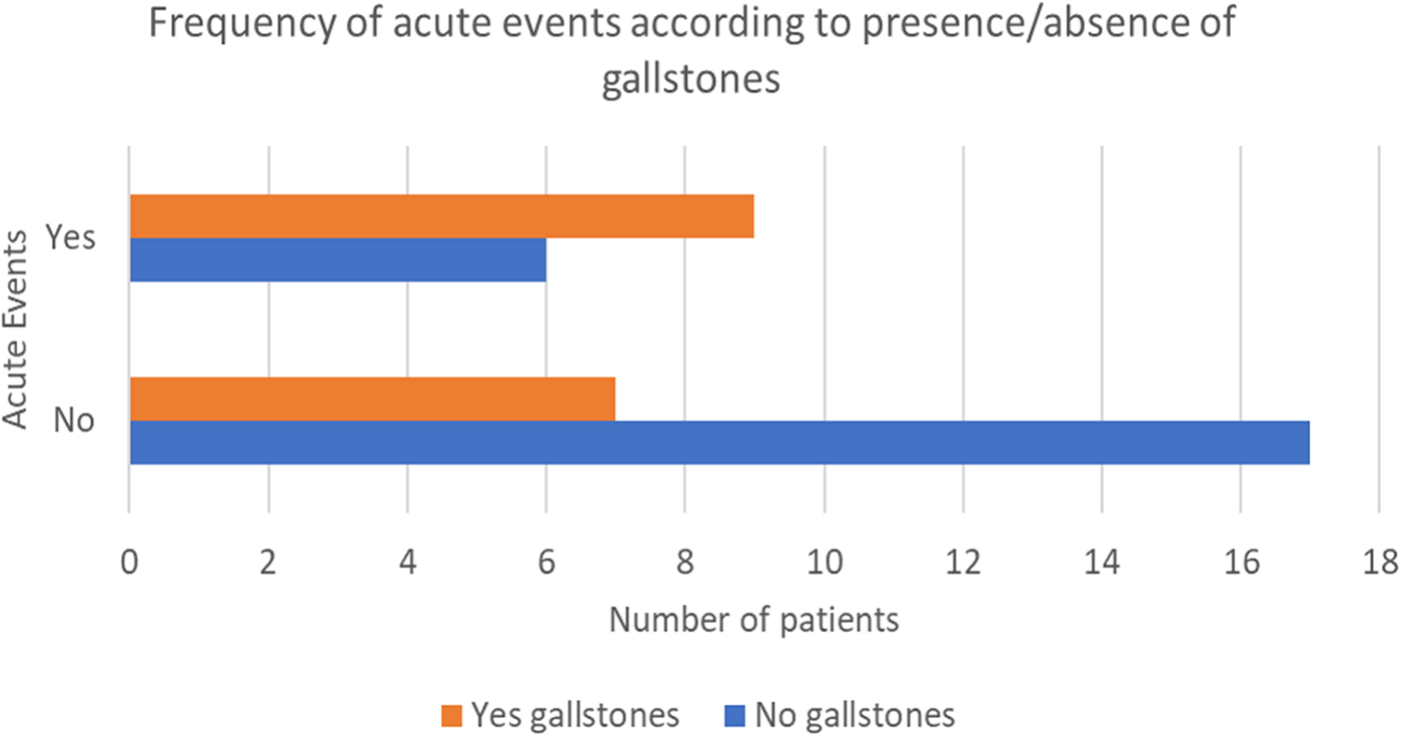
Frequency of acute events according to presence/absence of gallstones.

A total of 40 out of 42 patients underwent an abdominal ultrasound, which was performed at a mean age of 5 years and 7 months (±3 years and 10 months). 16/40 (40%) patients had gallstones, diagnosed at a mean age of 7 years and 3 months (±2 years and 2 months); in half of these patients cholelithiasis was found during the first abdominal ultrasound. 53.3% had a personal history of symptoms related to gallstones presence, like cholestatic jaundice or biliary cholic. Hemolytic crises and parvovirus infections were more frequent in patients with stones (53.6% vs. 26.1% and 63.6% vs. 28.6%, respectively) (Figure 1). Moreover, 56.3% needed at least one hospitalization for acute anemia or aplastic crisis due to Parvovirus B19 infection.

A total of 10 of 16 patients with cholelithiasis, underwent elective cholecystectomy, 72.7% of them with concomitant splenectomy; 54.5% had a history of jaundice or biliary cholic; 63.6% had been hospitalized for hemolytic crisis or Parvovirus B19 infection.

No statistical significance was found regarding steady state hematological parameters between patients with and without gallstones, (Table 2). However, all patients receiving cholecystectomy had severe or moderate phenotype. Overall, 2 patients had severe phenotype (transfusion dependent until splenectomy was performed), 12 were moderate, 28 were mild.

**Table 2:** Steady state hematological parameters in patients with and without gallstones.

Variable	No gallstones *N* = 24	Yes gallstones *N* = 16	*P*-Value
Red blood cells 10^12^/L (Mean ± SD)	3.57 ± 0.88	3.47 ± 0.71	0.73
Hemoglobin g/L (Mean ± SD)	103.48 ± 29.25	100.69 ± 19.20	0.92
Hematocrit (Mean ± SD)	30.49 ± 7.53	28.18 ± 5.74	0.52
Reticulocytes cells/ul (Mean ± SD)	279.47 ± 199.22	285.59 ± 156.82	0.72
Total bilirubin mg/dl (Mean ± SD)	6.31 ± 12.43	6.25 ± 3.33	0.027
gGT U/L (Mean ± SD)	52.00 ± 53.25	51.40 ± 53.47	0.83
ALP U/L (Mean ± SD)	208.00*	237.00*	
LDH U/L (Mean ± SD)	574.64 ± 402.31	564.60 ± 371.84	1.00
Aptoglobin mg/dl n.v. 30–200 (Mean ± SD)	17.47 ± 19.45	7.83 ± 0.41	0.45
AST mU/ml (Mean ± SD)	38.45 ± 13.59	39.75 ± 17.85	0.93
Ferritin ng/ml (Mean ± SD)	566.25 ± 593.87	293.58 ± 295.81	1.00

* Data was available for only one patient.

### Spleen complications

A total of 14/40 (35%) received indication for splenectomy, of whom 13 performed the surgery (one patient refused the intervention). Indications for splenectomy were transfusion dependence (*N* = 2), splenomegaly accompanied by recurrent acute events with severe anemia and transfusion need (*N* = 12). All patients received full vaccination schedule before splenectomy which was performed after 6 years of age. Ten patients underwent a total splenectomy, 3 a partial one. Eight patients had a simultaneous cholecystectomy. Among the remaining 6 patients, 4 did not have cholelithiasis while in 2 patients choledochal gallstones appeared after splenectomy.

### Follow up compliance

Thirty-five patients (83.3%) had compliance with clinic appointments, having at least one hematology visit per year; the remaining 7 patients did not respect the timing for visit recommended, and all these cases had a mild disease.

## Discussion

This is the first study on the clinical and laboratory characteristic and hepatobiliary complications of patients affected by hereditary spherocytosis living in Veneto Region after the implementation of the pediatric oncological and hematological regional network.

In all patients, the diagnostic suspect of HS was made in spoke Centers (1st or 2nd level Hospitals). The upper-level tests and the confirmation of diagnosis were made in the hub center. This confirms the importance of networking for rare hematological diseases (15–17).

None of the patients received the diagnosis of HS during the neonatal period, in spite of severity of symptoms experienced by some patients in the first month of life. Moreover, 46% of cases lacked information regarding clinical history in the newborn period. The need to perform education and training on hematological rare diseases in pediatric general initiatives. The above data, coupled with the 6 months of diagnostic delay to obtain the definitive diagnosis of HS, underscore the need to improve knowledge on HS in the hub-and-spoke network of general pediatricians, but also to improve the workflow and shipping of samples in the network.

When the two confirmatory tests were available, starting in 2017, time to diagnosis was reduced (from 6 months to 3.5 months ±6 months), due to the possibility of avoiding the bureaucracy of shipping samples outside the Region and the percentage of patients performing two confirmatory tests increased from 64.8% to 100%, when all the tests could be done in our Center. The local availability of the techniques and a better training of the laboratory staff in the framework of the EuroBloodNet network contributed to the optimizing diagnostic pathway, but educational events could improve diagnostic outcomes further.

A total of 16 of 40 patients (40%) had cholelithiasis, more than reported in a previous pediatric survey in Italy (11), with a mean age of appearance of 7 years and 4 months, earlier than described in other case series (18), in which gallstones were found at a median age between 8.7 and 13 years depending on the severity of the disease. Half of patients had gallstones at the first abdominal ultrasound (that is performed at a mean age of 5 years and 7 months), demonstrating that maybe mean age of lithiasis appearance could be lower. This fact suggests that in our population it could be useful to anticipate the first abdominal ultrasound, in order to avoid acute complications that happen more frequently in population with gallstones. Moreover, while the indications for splenectomy in severe spherocytosis are defined (13), those for cholecystectomy in asymptomatic patients need to be rediscussed taking into consideration phenotype severity, genetic variations and surgical techniques (19–23). Furthermore, in the ultrasound reports the liver and spleen dimensions and the lithiasis dimensions are not always described, making difficult the assess the efficacy of treatment in the long term. For this reason, a standardized method to evaluate ultrasound should be beneficial.

Our study is limited by the small number of patients and by its monocentric and retrospective nature, nevertheless it allows a first evaluation of the diagnostic pathway and the medium-long term biliary complications of HS in a pediatric cohort for which care within a specialized network is available. In the future, a prospective multicentric study, with the integration of genetic, metabolic and functional analysis, could aid in better characterizing phenotypic variability and personalize treatment (24–26). The recent development of the Rare Anemia Disorders European Epidemiological Platform (RADeep), an important initiative endorsed by ERN-EuroBloodNet, for a European approach for the standardized collection of data regarding the main clinical complications of rare anemias points toward this direction (27).

## Conclusions

Our study has unveiled certain shortcomings in the diagnostic process and treatment of HS. There is room for enhancement in the suspicion index of HS, which could lead to a decrease in diagnostic delays through educational initiatives organized within the hub-and-spoke network. Furthermore, a modification in hepatobiliary follow-up practices is suggested, including the implementation of the initial abdominal ultrasound at the age of 3. The advent of new molecular techniques, such as NGS, now at our disposal, offers the potential to delve into the genetic factors contributing to phenotypic variability, thereby enabling tailored treatments of follow-up plans for specific patient subgroups.

## Data Availability

The raw data supporting the conclusions of this article will be made available by the authors, without undue reservation.
